# 1,5-Dimethyl-3-oxo-2-phenyl-2,3-dihydro-1*H*-pyrazol-4-aminium bromide monohydrate

**DOI:** 10.1107/S1600536812023550

**Published:** 2012-05-31

**Authors:** Yan-Yun Yang, Liang Xu, Ting-Guo Kang, Ting Chen, Ping Wu

**Affiliations:** a Liaoning University of Traditional Chinese Medicine, Dalian 116600, People’s Republic of China

## Abstract

In the title hydrated mol­ecular salt, C_11_H_14_N_3_O^+^·Br^−^·H_2_O, the Br^−^ anion is split and appears as two independent half-occupied Br^−^ anions on twofold rotation axes. The dihedral angle between the phenyl ring and the mean plane of the 2,3-dihydro-1*H*-pyrazole ring (r.m.s. devation = 0.014 Å) is 62.43 (7)°. In the crystal, the components are connected *via* O—H⋯Br and N—H⋯O hydrogen bonds to form a one-dimensional polymeric structure propagating along [001].

## Related literature

For general background on pyrazolone derivatives, see: Casas *et al.* (2007 ▶); Jain *et al.* (2003 ▶); Zhang *et al.* (2008 ▶). For related structures, see: Chitradevi *et al.* (2009 ▶); Murtaza *et al.*(2011 ▶). For bond-length data, see: Allen *et al.* (1987 ▶).
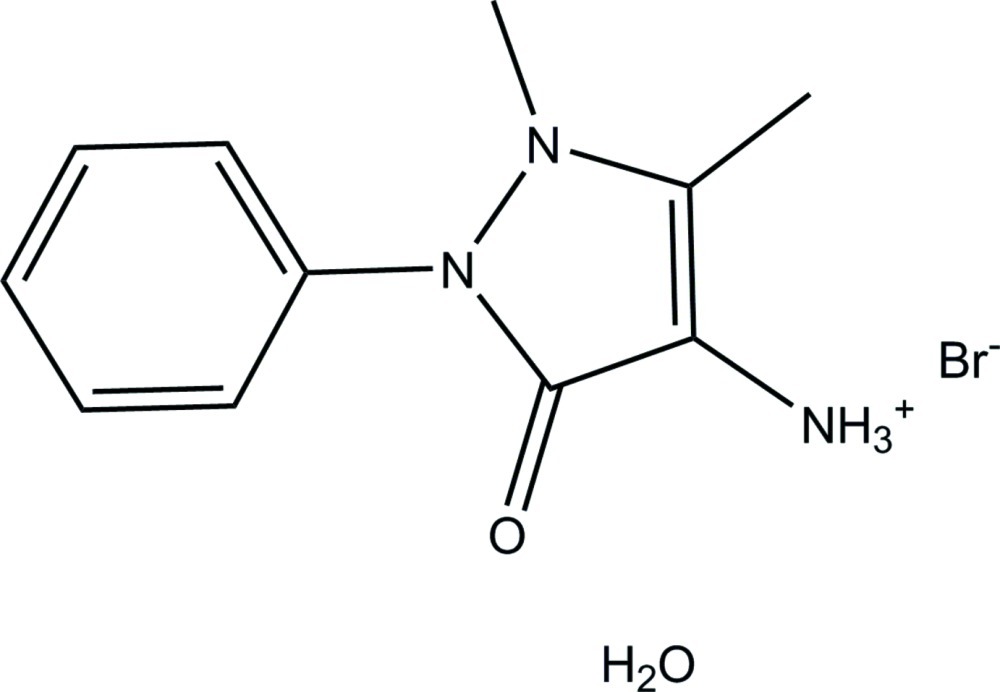


## Experimental

### 

#### Crystal data

C_11_H_14_N_3_O^+^·Br^−^·H_2_O
*M* *_r_* = 302.17
Monoclinic, 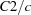

*a* = 14.9080 (19) Å
*b* = 15.3961 (19) Å
*c* = 11.1501 (14) Å
β = 93.657 (2)°
*V* = 2554.0 (6) Å^3^
*Z* = 8
Mo *K*α radiation
μ = 3.21 mm^−1^
*T* = 296 K
0.22 × 0.20 × 0.18 mm

#### Data collection

Bruker SMART CCD diffractometer
Absorption correction: multi-scan (*SADABS*; Sheldrick, 1996 ▶) *T*_min_ = 0.489, *T*_max_ = 0.556
11995 measured reflections
3222 independent reflections
2081 reflections with *I* > 2σ(*I*)
*R*_int_ = 0.044

#### Refinement

*R*[*F* ^2^ > 2σ(*F* ^2^)] = 0.039
*wR*(*F* ^2^) = 0.108
*S* = 1.04
3222 reflections
155 parameters
H-atom parameters constrained
Δρ_max_ = 0.48 e Å^−3^
Δρ_min_ = −0.40 e Å^−3^

### 

Data collection: *SMART* (Bruker, 1998 ▶); cell refinement: *SAINT* (Bruker, 1998 ▶); data reduction: *SAINT*; program(s) used to solve structure: *SHELXS97* (Sheldrick, 2008 ▶); program(s) used to refine structure: *SHELXL97* (Sheldrick, 2008 ▶); molecular graphics: *SHELXTL* (Sheldrick, 2008 ▶); software used to prepare material for publication: *SHELXTL*.

## Supplementary Material

Crystal structure: contains datablock(s) I, global. DOI: 10.1107/S1600536812023550/su2432sup1.cif

Structure factors: contains datablock(s) I. DOI: 10.1107/S1600536812023550/su2432Isup2.hkl

Supplementary material file. DOI: 10.1107/S1600536812023550/su2432Isup3.cml

Additional supplementary materials:  crystallographic information; 3D view; checkCIF report

## Figures and Tables

**Table 1:** Hydrogen-bond geometry (Å, °)

*D*—H⋯*A*	*D*—H	H⋯*A*	*D*⋯*A*	*D*—H⋯*A*
O1*W*—H1*WA*⋯Br1	0.85	2.51	3.360 (2)	173
O1*W*—H1*WB*⋯Br2	0.85	2.52	3.371 (3)	173
N3—H3*B*⋯O1^i^	0.89	1.89	2.692 (3)	150
N3—H3*C*⋯O1*W*	0.89	2.55	3.321 (3)	146
N3—H3*C*⋯O1^ii^	0.89	2.37	3.004 (3)	129
N3—H3*D*⋯O1*W*^ii^	0.89	1.99	2.817 (4)	153

Symmetry codes: (i) 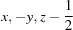; (ii) 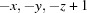.

**Fig. 1.**
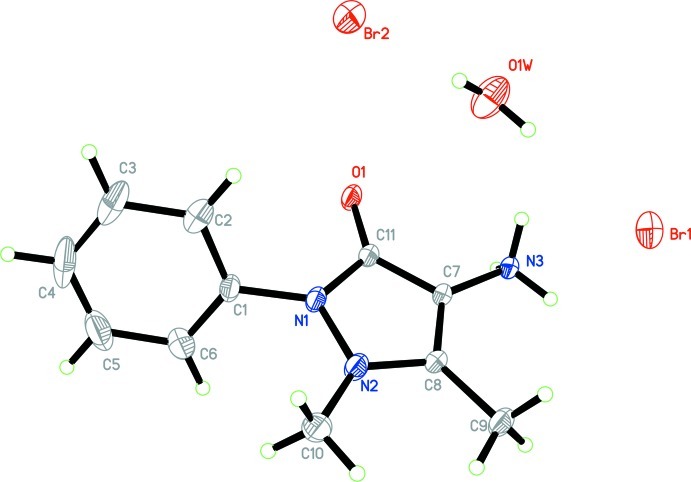
The molecular structure of the title compound with the atom numbering. The displacement ellipsoids are drawn at the 30% probability level.

**Fig. 2.**
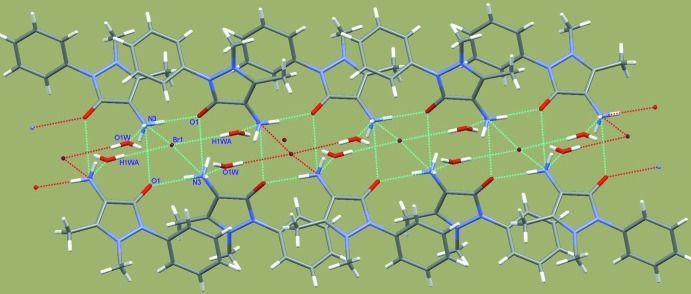
A view along the c axis of the crystal packing of the title compound. H-bonds are shown as dashed lines; see Table 1 for details.

## supplementary crystallographic information

### Comment

4-Aminoantipyrene, which contains a pyrazolone ring, is an important compound in the class of analgesic agents used in otic solutions in combination with other analgesics such as benzocaine and phenylephrine (Jain *et al.*, 2003). Pyrazolone is a five-membered lactam ring compound containing two N atoms and a ketone in the same molecule. Such pyrazolone derivatives form a very important class of heterocycles due to their properties and applications (Casas *et al.*, 2007; Zhang *et al.*, 2008). We report herein on the synthesis and crystal structure of the title compound.

The asymmetric unit of title compound, Fig. 1, consists of three components: a 1,5-dimethyl-3-oxo-2-phenyl-2,3-dihydro-1*H*-pyrazol-4-aminium cation, a bromide ion and a water molecule. The Br anion is split and appears as two independent half-occupied Br anions, Br1 and Br2, on two-fold rotation axes. In cation, the phenyl ring A (C1–C6) and the 2,3-dihydro-1*H*-pyrazole ring B (N1/N2/C7/C8/C11) are planar with r.m.s. deviations of 0.019 and 0.014 Å. The dihedral angle between A/B is 62.43 (7)°. The attached atoms O1, N3, C9 and C10 are at a distance of 0.030 (3), 0.053 (3), 0.130 (3) and 0.140 (3) Å respectively, from the mean plane of B. The bond lengths (Allen *et al.*, 1987) and angles are within normal ranges. The crystal structure of some similar compounds have been reported (Chitradevi *et al.*, 2009; Murtaza *et al.*, 2011).

In the crystal, the various components are connected by N—H···O and O—H···Br hydrogen bonds (Table 1 and Fig. 2) to form an infinite one-dimensional arrangement parallel to [001].

### Experimental

4-Aminoantipyrine (0.203 g, 1.0 mmol) and dibromomethane (0.173 g, 1.0 mmol) were dissolved in water (15 ml). The mixture was refluxed for 3 h and then the solvent was evaporated on rotary evaporator to almost dryness. The crude product was recrystallized from water yielding block-like yellow crystals of the title compound.

### Refinement

The H atoms were included calculated positions and treated as riding atoms: O—H = 0.85 Å, N—H = 0.89 Å, C—H = 0.93–0.96 Å, with *U*_iso_(H) = *x × U*eq(C,N,O), where *x* = 1.5 for CH_3_ and NH_3_ H atoms and = 1.2 for other H-atoms.

### Crystal data

**Table:**

C_11_H_14_N_3_O^+^·Br^−^·H_2_O	*F*(000) = 1232
*M**_r_* = 302.17	*D*_x_ = 1.572 Mg m^−^^3^
Monoclinic, *C*2/*c*	Mo *K*α radiation, λ = 0.71073 Å
Hall symbol: -C 2yc	Cell parameters from 2078 reflections
*a* = 14.9080 (19) Å	θ = 2.7–23.6°
*b* = 15.3961 (19) Å	µ = 3.21 mm^−^^1^
*c* = 11.1501 (14) Å	*T* = 296 K
β = 93.657 (2)°	Block, yellow
*V* = 2554.0 (6) Å^3^	0.22 × 0.20 × 0.18 mm
*Z* = 8	

### Data collection

**Table:**

Bruker SMART CCD diffractometer	3222 independent reflections
Radiation source: fine-focus sealed tube	2081 reflections with *I* > 2σ(*I*)
Graphite monochromator	*R*_int_ = 0.044
ω scans	θ_max_ = 28.4°, θ_min_ = 2.7°
Absorption correction: multi-scan (*SADABS*; Sheldrick, 1996)	*h* = −19→19
*T*_min_ = 0.489, *T*_max_ = 0.556	*k* = −20→20
11995 measured reflections	*l* = −14→14

### Refinement

**Table:**

Refinement on *F*^2^	Primary atom site location: structure-invariant direct methods
Least-squares matrix: full	Secondary atom site location: difference Fourier map
*R*[*F*^2^ > 2σ(*F*^2^)] = 0.039	Hydrogen site location: inferred from neighbouring sites
*wR*(*F*^2^) = 0.108	H-atom parameters constrained
*S* = 1.04	*w* = 1/[σ^2^(*F*_o_^2^) + (0.0493*P*)^2^ + 2.161*P*] where *P* = (*F*_o_^2^ + 2*F*_c_^2^)/3
3222 reflections	(Δ/σ)_max_ < 0.001
155 parameters	Δρ_max_ = 0.48 e Å^−^^3^
0 restraints	Δρ_min_ = −0.40 e Å^−^^3^

### Special details

**Table:**

Geometry. Bond distances, angles etc. have been calculated using the rounded fractional coordinates. All su's are estimated from the variances of the (full) variance-covariance matrix. The cell esds are taken into account in the estimation of distances, angles and torsion angles
Refinement. Refinement of *F*^2^ against ALL reflections. The weighted *R*-factor *wR* and goodness of fit *S* are based on *F*^2^, conventional *R*-factors *R* are based on *F*, with *F* set to zero for negative *F*^2^. The threshold expression of *F*^2^ > σ(*F*^2^) is used only for calculating *R*-factors(gt) *etc*. and is not relevant to the choice of reflections for refinement. *R*-factors based on *F*^2^ are statistically about twice as large as those based on *F*, and *R*-factors based on ALL data will be even larger.

### Fractional atomic coordinates and isotropic or equivalent isotropic displacement parameters (Å^2^)

**Table:**

	*x*	*y*	*z*	*U*_iso_*/*U*_eq_	
O1	0.11377 (13)	0.01137 (15)	0.64912 (16)	0.0377 (6)	
N1	0.24203 (15)	0.09233 (18)	0.61672 (19)	0.0346 (8)	
N2	0.28247 (16)	0.12321 (17)	0.5164 (2)	0.0353 (8)	
N3	0.08569 (14)	0.01740 (16)	0.38186 (18)	0.0286 (7)	
C1	0.2831 (2)	0.0971 (2)	0.7361 (2)	0.0330 (9)	
C2	0.2374 (2)	0.1400 (2)	0.8221 (3)	0.0437 (10)	
C3	0.2752 (3)	0.1419 (3)	0.9393 (3)	0.0630 (16)	
C4	0.3572 (4)	0.1054 (3)	0.9670 (3)	0.080 (2)	
C5	0.4009 (3)	0.0627 (4)	0.8813 (4)	0.0781 (18)	
C6	0.3637 (2)	0.0573 (3)	0.7639 (3)	0.0551 (13)	
C7	0.15821 (17)	0.05670 (17)	0.4544 (2)	0.0256 (8)	
C8	0.22945 (18)	0.10325 (18)	0.4178 (2)	0.0275 (8)	
C9	0.2493 (2)	0.1347 (2)	0.2962 (3)	0.0391 (10)	
C10	0.3605 (2)	0.1798 (2)	0.5249 (3)	0.0466 (10)	
C11	0.16456 (18)	0.04939 (18)	0.5808 (2)	0.0281 (8)	
Br1	0.00000	0.19903 (3)	0.25000	0.0427 (2)	
Br2	0.00000	0.29956 (4)	0.75000	0.0536 (2)	
O1W	−0.04198 (19)	0.14894 (18)	0.5343 (2)	0.0651 (10)	
H2A	0.18260	0.16700	0.80220	0.0530*	
H3A	0.24430	0.16840	0.99940	0.0760*	
H3B	0.09260	0.02850	0.30470	0.0430*	
H3C	0.03360	0.03920	0.40250	0.0430*	
H3D	0.08620	−0.03980	0.39370	0.0430*	
H4A	0.38340	0.10970	1.04480	0.0950*	
H5A	0.45620	0.03670	0.90140	0.0940*	
H6A	0.39310	0.02730	0.70560	0.0660*	
H9A	0.30540	0.16560	0.30100	0.0590*	
H9B	0.20210	0.17280	0.26610	0.0590*	
H9C	0.25330	0.08610	0.24290	0.0590*	
H10A	0.37780	0.19340	0.44560	0.0700*	
H10B	0.40920	0.15110	0.56910	0.0700*	
H10C	0.34590	0.23250	0.56550	0.0700*	
H1WA	−0.02990	0.16590	0.46460	0.0780*	
H1WB	−0.02990	0.18990	0.58390	0.0780*	

### Atomic displacement parameters (Å^2^)

**Table:**

	*U*^11^	*U*^22^	*U*^33^	*U*^12^	*U*^13^	*U*^23^
O1	0.0326 (10)	0.0618 (14)	0.0186 (9)	−0.0133 (10)	0.0006 (8)	0.0052 (10)
N1	0.0309 (13)	0.0554 (16)	0.0174 (10)	−0.0125 (12)	0.0004 (9)	0.0000 (11)
N2	0.0365 (14)	0.0473 (15)	0.0222 (11)	−0.0144 (11)	0.0023 (10)	0.0009 (11)
N3	0.0255 (11)	0.0427 (14)	0.0174 (10)	−0.0025 (10)	−0.0004 (8)	−0.0018 (10)
C1	0.0337 (14)	0.0450 (18)	0.0199 (13)	−0.0118 (14)	−0.0022 (11)	0.0009 (13)
C2	0.062 (2)	0.0393 (18)	0.0301 (16)	−0.0069 (16)	0.0059 (15)	−0.0011 (14)
C3	0.106 (4)	0.056 (2)	0.0272 (17)	−0.026 (2)	0.006 (2)	−0.0081 (17)
C4	0.105 (4)	0.098 (4)	0.032 (2)	−0.057 (3)	−0.026 (2)	0.014 (2)
C5	0.047 (2)	0.126 (4)	0.058 (3)	−0.022 (2)	−0.023 (2)	0.028 (3)
C6	0.0382 (18)	0.082 (3)	0.044 (2)	−0.0042 (18)	−0.0058 (15)	0.0046 (18)
C7	0.0251 (13)	0.0337 (14)	0.0180 (12)	−0.0002 (11)	0.0006 (9)	−0.0022 (11)
C8	0.0283 (14)	0.0347 (15)	0.0197 (12)	−0.0011 (12)	0.0032 (10)	0.0006 (11)
C9	0.0439 (18)	0.0505 (19)	0.0232 (13)	−0.0086 (14)	0.0043 (12)	0.0062 (14)
C10	0.0380 (17)	0.065 (2)	0.0369 (17)	−0.0226 (16)	0.0040 (13)	−0.0049 (16)
C11	0.0264 (13)	0.0388 (16)	0.0191 (12)	−0.0037 (12)	0.0011 (10)	0.0002 (12)
Br1	0.0493 (3)	0.0400 (3)	0.0371 (2)	0.0000	−0.0094 (2)	0.0000
Br2	0.0436 (3)	0.0828 (4)	0.0347 (3)	0.0000	0.0055 (2)	0.0000
O1W	0.097 (2)	0.0533 (16)	0.0461 (14)	−0.0197 (15)	0.0132 (14)	0.0000 (13)

### Geometric parameters (Å, º)

**Table:**

O1—C11	1.253 (3)	C4—C5	1.360 (7)
O1W—H1WB	0.8500	C5—C6	1.391 (6)
O1W—H1WA	0.8500	C7—C8	1.365 (4)
N1—C11	1.368 (4)	C7—C11	1.411 (3)
N1—C1	1.431 (3)	C8—C9	1.487 (4)
N1—N2	1.388 (3)	C2—H2A	0.9300
N2—C8	1.348 (3)	C3—H3A	0.9300
N2—C10	1.452 (4)	C4—H4A	0.9300
N3—C7	1.441 (3)	C5—H5A	0.9300
N3—H3C	0.8900	C6—H6A	0.9300
N3—H3D	0.8900	C9—H9B	0.9600
N3—H3B	0.8900	C9—H9C	0.9600
C1—C2	1.380 (4)	C9—H9A	0.9600
C1—C6	1.367 (5)	C10—H10C	0.9600
C2—C3	1.390 (5)	C10—H10A	0.9600
C3—C4	1.363 (7)	C10—H10B	0.9600
			
H1WA—O1W—H1WB	109.00	N2—C8—C7	107.6 (2)
N2—N1—C1	123.4 (2)	N1—C11—C7	104.8 (2)
N2—N1—C11	109.4 (2)	O1—C11—C7	129.8 (2)
C1—N1—C11	126.9 (2)	O1—C11—N1	125.4 (2)
N1—N2—C8	108.5 (2)	C3—C2—H2A	121.00
N1—N2—C10	122.7 (2)	C1—C2—H2A	121.00
C8—N2—C10	128.0 (2)	C2—C3—H3A	120.00
C7—N3—H3D	109.00	C4—C3—H3A	120.00
C7—N3—H3B	109.00	C5—C4—H4A	120.00
C7—N3—H3C	109.00	C3—C4—H4A	120.00
H3C—N3—H3D	110.00	C4—C5—H5A	120.00
H3B—N3—H3C	110.00	C6—C5—H5A	120.00
H3B—N3—H3D	109.00	C5—C6—H6A	121.00
N1—C1—C6	120.4 (3)	C1—C6—H6A	121.00
N1—C1—C2	118.0 (3)	C8—C9—H9A	109.00
C2—C1—C6	121.5 (3)	C8—C9—H9B	109.00
C1—C2—C3	118.4 (3)	H9A—C9—H9B	109.00
C2—C3—C4	120.6 (3)	H9A—C9—H9C	109.00
C3—C4—C5	120.2 (4)	C8—C9—H9C	110.00
C4—C5—C6	120.7 (4)	H9B—C9—H9C	109.00
C1—C6—C5	118.6 (3)	N2—C10—H10B	110.00
N3—C7—C11	121.8 (2)	N2—C10—H10C	109.00
C8—C7—C11	109.7 (2)	N2—C10—H10A	109.00
N3—C7—C8	128.5 (2)	H10A—C10—H10C	109.00
N2—C8—C9	121.9 (2)	H10B—C10—H10C	109.00
C7—C8—C9	130.3 (2)	H10A—C10—H10B	110.00
			
C1—N1—N2—C8	175.6 (3)	N1—C1—C2—C3	177.5 (3)
C1—N1—N2—C10	−13.9 (4)	C6—C1—C2—C3	−0.2 (5)
C11—N1—N2—C8	2.5 (3)	N1—C1—C6—C5	−179.3 (4)
C11—N1—N2—C10	173.0 (3)	C2—C1—C6—C5	−1.8 (6)
N2—N1—C1—C2	122.6 (3)	C1—C2—C3—C4	2.8 (6)
C11—N1—C1—C2	−65.5 (4)	C2—C3—C4—C5	−3.4 (7)
N2—N1—C1—C6	−59.8 (4)	C3—C4—C5—C6	1.5 (8)
C11—N1—C1—C6	112.1 (4)	C4—C5—C6—C1	1.1 (7)
N2—N1—C11—C7	−1.1 (3)	N3—C7—C8—C9	6.8 (5)
C1—N1—C11—C7	−174.0 (3)	C11—C7—C8—N2	2.1 (3)
N2—N1—C11—O1	177.5 (3)	C11—C7—C8—C9	−174.3 (3)
C1—N1—C11—O1	4.7 (5)	N3—C7—C11—O1	−0.1 (5)
C10—N2—C8—C9	4.1 (5)	N3—C7—C11—N1	178.5 (2)
C10—N2—C8—C7	−172.6 (3)	C8—C7—C11—O1	−179.2 (3)
N1—N2—C8—C9	174.0 (3)	C8—C7—C11—N1	−0.6 (3)
N1—N2—C8—C7	−2.7 (3)	N3—C7—C8—N2	−176.9 (3)

### Hydrogen-bond geometry (Å, º)

**Table:**

*D*—H···*A*	*D*—H	H···*A*	*D*···*A*	*D*—H···*A*
O1*W*—H1*WA*···Br1	0.85	2.51	3.360 (2)	173
O1*W*—H1*WB*···Br2	0.85	2.52	3.371 (3)	173
N3—H3*B*···O1^i^	0.89	1.89	2.692 (3)	150
N3—H3*C*···O1*W*	0.89	2.55	3.321 (3)	146
N3—H3*C*···O1^ii^	0.89	2.37	3.004 (3)	129
N3—H3*D*···O1*W*^ii^	0.89	1.99	2.817 (4)	153

Symmetry codes: (i) *x*, −*y*, *z*−1/2; (ii) −*x*, −*y*, −*z*+1.
